# Nucleoporins cooperate with Polycomb silencers to promote transcriptional repression and repair at DNA double strand breaks

**DOI:** 10.21203/rs.3.rs-4680344/v1

**Published:** 2024-07-16

**Authors:** Hongseon Song*, Yubin Bae*, Sangin Kim*, Dante Deascanis*, Yujin Lee, Gergely Rona, Ethan Lane, Seoyeong Lee, Sujung Kim, Michele Pagano, Kyungjae Myung, Younghoon Kee

## Abstract

DNA Double-strand breaks (DSBs) are harmful lesions and major sources of genomic instability. Studies have suggested that DSBs induce local transcriptional silencing that consequently promotes genomic stability. Several factors have been proposed to actively participate in this process, including ATM and Polycomb repressive complex 1 (PRC1). Here we found that disrupting PRC1 clustering disrupts DSB-induced gene silencing. Interactome analysis of PHC2, a PRC1 subunit that promotes the formation of the Polycomb body, found several nucleoporins that constitute the Nuclear Pore Complex (NPC). Similar to PHC2, depleting the nucleoporins also disrupted the DSB-induced gene silencing. We found that some of these nucleoporins, such as NUP107 and NUP43, which are members of the Y-complex of NPC, localize to DSB sites. These nucleoporin-enriched DSBs were distant from the nuclear periphery. The presence of nucleoporins and PHC2 at DSB regions were inter-dependent, suggesting that they act cooperatively in the DSB-induced gene silencing. We further found two structural components within NUP107 to be necessary for the transcriptional repression at DSBs: ATM/ATR-mediated phosphorylation at Serine37 residue within the N-terminal disordered tail, and the NUP133-binding surface at the C-terminus. These results provide a new functional interplay among nucleoporins, ATM and the Polycomb proteins in the DSB metabolism, and underscore their emerging roles in genome stability maintenance. *Hongseon Song, Yubin Bae, Sangin Kim, and Dante Deascanis contributed equally to this work.

## Introduction

Various genetic mutations, chemicals (DNA crosslinking agents, replication fork stalling agents) or intracellular hazards (e.g. Reactive oxygen species) can induce DNA double-strand breaks (DSBs). Inappropriate handling of DSBs can lead to loss of genetic materials, chromosome translocations, or aneuploidy [1]. To safeguard the genomic integrity, cells employ several types of DSB repair mechanisms including homologous recombination (HR) and non-homologous end-joining (NHEJ). The efficiency of the DNA repair processes is promoted by chromatin modification or remodeling, dictated by DNA damage response kinases such as ATM (Ataxia-telangiectasia mutated) and ATR (Ataxia telangiectasia and Rad3-related). A series of studies have also highlighted evidence of DSB mobility and relocation; in the studies primarily done in yeast, chromatin area bearing DSBs acquire increased mobility and relocate to the nuclear periphery [2,3]. Studies mainly done in yeast and drosophila showed that tethering of DSBs to the nuclear periphery, via interacting with nuclear lamina or NPC (nuclear pore complex), promotes genomic integrity (reviewed in [3,4]). Some evidence indicated that DSBs in mammalian cells also relocate – for instance, dysfunctional telomeres move to the nuclear periphery [5] and DSBs in ribosomal DNA relocate to the nucleolar periphery [6]. More recently, DSBs interact with nuclear envelope components via inward invagination of the nuclear membrane [7].

Although it is generally thought that such transient translocation generates a condition that promotes appropriate DNA repair processes, a proposed mechanism for the tethering of the nuclear periphery components to DSBs to stimulate genomic integrity remains to be fully elucidated. One of the early responses to DSB formation is a transcriptional repression around the DSB sites. The transcriptional shut-off is an actively controlled process, which is regulated by ATM [8], chromatin organizers such as Cohesin [9], Polycomb proteins [10-12], PBAF (Polybromo, Brg1-associated factors) [12,13], NuRD (Nucleosome remodeling and deacetylase) complex [14] as well as direct RNA polymerase II regulation by NELFE [15], DYRK1B kinase [16], histone chaperones FACT (Facilitates chromatin transcription) [17] and RSF (Remodeling and spacing factor) [18]. However, whether this transcriptional regulation is coordinated within the context of the DSB relocation to the nuclear periphery is unknown.

Herein, we describe a new role of the Nuclear Pore Complex (NPC) in the DSB-induced transcriptional repression. We first found that PHC2 (Polyhomeotic Homolog 2), an integral subunit of PRC1 (Polycomb Repressive Complex 1) that promotes PRC1 clustering and Polycomb body formation [19] is required for the transcription repression at DSBs. Proteomic analysis of PHC2 interactors found several members (nucleoporins) of NPC. Depleting the nucleoporin proteins (e.g. NUP107) had similar effects on transcription repression at DSBs. Some of these nucleoporins localize to DSB sites, but the locations of these overlapped regions were distant from the lamina-stained nuclear periphery. The presence of Polycomb proteins and nucleoporins at DSBs were inter-dependent, and depleting either PHC2 or NUP107 impaired HR repair capacity and genomic stability. We found that ATM/ATR-mediated phosphorylation of the S37 residue within the NUP107 disordered tail to be an important contributor to both DSB-induced gene silencing and HR repair. We also found that integrity of the Y-complex is required for this process. Overall, these data uncovered a previously unknown role of nucleoporins in DSB processing, and reinforced the concept that nuclear pore components promote genomic stability.

## Materials and Methods

*Cell lines, plasmids and chemicals*

HeLa, HCT116, and U2OS, U2OS Ptuner263 cells (provided by Roger Greenberg), and DR-GFP cells (provided by Maria Jasin) cells were cultured in Dulbecco's Modified Eagles Medium (DMEM) supplemented with 10% fetal bovine serum (FBS) and L-glutamine (Conc). Cells were maintained in respective media containing penicillin and streptomycin and incubated at 37°C in 5% CO2. The The pBABE-HA-ER-IPpoI plasmid was purchased from Addgene. The plasmids were transfected into HCT116 cells and the single cell clones stably expressing HA-ER-I-Ppo1 were isolated. 3xFLAG-NUP107 wild type and mutations (S37A, ΔN140, D831A, and Y889C) were generated by VectorBuilder Inc. GFP-RNF2, expressed from a retroviral vector (pBABE.puro), was described earlier[10]. PHC2 cDNA (isoform b) was isolated from HeLa cells and subcloned into the pBABE and GFP vectors. Transfection of plasmid DNA was performed using Turbofect (Thermo Fisher Scientific) and X-tremeGENE™ HP (Roche). A list of chemicals used in the study can be found in Supplementary Methods and Materials.

*Western blots and antibodies*

Cells were first harvested by washing with 1x Phosphate-buffered saline (PBS) and scraped with polyethylene scraper. Cell pellets were lysed and resuspended in 2x lysis buffer (0.5% SDS, 0.5 M Tris-HCl). The samples were boiled at 95°C for 10 minutes and then spun down for 1 minute at 14000 rpm to pellet cell debris. The protein concentration of each sample is determined using Protein Assay Dye (Bio-Rad) and recording optical density measurements at 595 nm. The resulting optical density measurements were compared to a standard curve of bovine serum albumin (BSA). 2x Laemmli buffer (Bio-Rad) was used to lyse the samples before boiling at 95°C for 3 minutes and centrifuged at 14000 rpm for 1 minutes. A list of antibodies used in this study can be found in Supplementary Methods and Materials.

*RNAi*

Cells were cultured in penicillin and streptomycin free media and transfected once with 20nM siRNA (final concentration) using the RNAiMAX (Invitrogen) reagent following the manufacturer's protocol. A list of all siRNAs used in this study can be found in Supplementary Methods and Materials.

*Immunofluorescence and image quantification*

Cells were seeded onto coverslips in 12-well plates and cultured in penicillin and streptomycin free media. For fixation, media was removed from the wells and washed twice with ice-cold 1X PBS and then fixed to coverslips for 15 minutes in the dark using 4% paraformaldehyde (PFA) (diluted in 1X PBS). Cells were washed twice with ice cold 1X PBS, permeabilized with 0.25% Triton X-100 in PBS for 20 minutes in the dark, and then washed twice with ice cold 1x PBS. Primary antibodies were diluted in 3% BSA and incubated on the coverslips overnight in the dark at 4°C. Cells were then washed with 0.05% Triton X in PBS and incubated with Alexa Fluor-conjugated secondary antibodies for 1 hour at room temperature. Cells were then washed with 0.05% Triton X in PBS. Coverslips were then mounted on microscope slides using the Vectashield mounting medium containing DAPI (Vector Laboratories Inc). Images were taken using the Zeiss Axiovert 200 microscope equipped with a Perkin Elmer ERS spinning disk confocal imager and a 63x/1.45NA oil objective using Volocity software (Perkin Elmer) or acquired by a Leica DMi8 microscope with Leica Application Suite (LAS X) software. All fluorescent images were quantified using ImageJ software (refer to each section for more detail according to each assay). Detailed procedures for image acquisition, UVC microfilter method, and pTUNER263 assay procedures are described in Supplementary Methods and Materials.

*3D image analysis*

HeLa cells stably expressing 3xFLAG-NUP107 were locally irradiated by UV 10J/$m^2$ through a 0.5μm microporefilter. Cells on coverslips were prepared as described in the '*Immunofluorescence and image quantification*'. 3D images and videos are acquired by Leica DMi8 microscope, and Z-stack images were processed with Leica Application Suite (LAS X) software for generating 3D images and videos.

*Microirradiation*

Laser-induced DNA damage induction and live-cell imaging were essentially performed as previously [10]. Briefly, ~50,000 U2OS cells stably expressing EGFP-RNF2, were plated per well on a four-well Lab-Tek II chambered number 1.5 borosilicate cover glass for 24 hours before imaging. RNAi transfections were performed approximately 2 days prior to microscopy using two rounds of silencing with the indicated siRNAs. Cells were pre-sensitized with 10 μM BrdU (Merck) for 24 hours. For imaging, on the day of data collection, cells were incubated in FluoroBrite DMEM supplemented with 10% FBS, 25 mM HEPES (Sigma-Aldrich), and 1% sodium pyruvate (Gibco). Imaging was performed using a DeltaVision Elite inverted microscope system (Applied Precision), using a 60× oil objective (1.42 NA) from Olympus. Excitation was achieved with a 7 Color Combined Insight solid state illumination system equipped with a polychroic beam splitter with filter sets to support GFP (ex.: 475/28 nm, em.: 525/50 nm) and mCherry (ex.: 575/25 nm, em.: 632/60 nm). Images were acquired using a CoolSNAP HQ2 camera. DNA damage was generated using a 405-nm, 50-mW laser at 100% power for 0.75 s. One pre-laser image was recorded, and the interval of post-laser images was 5 seconds for 5 minutes recordings. Recruitment intensity was analyzed using a macro written for ImageJ that calculated the ratio of intensity of a defined laser spot A to the adjacent area B after subtraction of the background intensity of an unpopulated area of image C [10]. Thus, the relative fluorescent unit (RFU) for each data collection point was calculated by the equation RFU = (A − C)/(B − C). In instances where fluorescent recruitment was not detected, the coordinates of A were determined using laser coordinates recorded in the data log file. The mean values and standard errors from several cells and live-cell imaging time courses were computed for each time point using GraphPad Prism software.

*5-EU-click chemistry*

Cells were labeled with 1 mM 5-EU after irradiation for 1 hours. After 5-EU labeling, a click reaction was performed using the Click-iT® EdU Imaging kit (C10643, Thermo Fisher Scientific), according to the manufacturer's instructions. Briefly, cells were fixed with 4% PFA for 20 minutes at room temperature and then rinsed with 1X cold-PBS. Cells were then extracted with cytoskeleton (CSK) buffer (10 mM PIPES pH 6.8, 100 mM NaCl, 300 mM sucrose, 3 mM $MgCl_2$, 1 mM EGTA and 0.5% Triton X-100™) for 10 minutes on ice. Cells were then fixed again with 4% PFA for 20 minutes at room temperature and subjected to immunostaining with γH2AX antibody.

*I-SceI-induced HR assay*

I-SceI-induced HR assay was performed using the U2OS-based HR reporter cell lines (DR-GFP) as described previously[20]. Briefly, reporter cells were plated on a 12-well plate at $1\times10^5$ cells/well. The following day, cells were transfected with 20 nM siRNA. After 24 hours, cells were transfected again with 0.5 μg of either pCAGGS-I-SceI (called pCBASce) or empty pCAGGS-BSKX vector, and 0.1 μg of dsRed expression vector. Two days after I-SceI transfection, cells were analyzed using a BD FACSVerse™ flow cytometer with BD FACSuite™ software (BD Biosciences). Repair efficiency was calculated by dividing the percentage of GFP-positive cells to the percentage of dsRed-positive cells. Data analysis was done using Flowjo software.

*I-Ppo1 nuclease Induced DSBs*

Stably pBABE-HA-ER-IppoI expressing HCT116 were seeded into 12-well plates and treated with the indicated siRNAs for 48 hours. 4-OHT was added into the culture media (final concentration: 2 μM) 3hours before fixation. After treatment, cells were fixed and stained following a previously described protocol (refer to Immunofluorescence and Image Quantification in methodology).

*Immunoprecipitation and mass spectrometry analysis*

3XFLAG-NUP107 knock-in HeLa cells were cultured in penicillin and streptomycin free media in 6 cm dishes. The following day, cells were transfected with either FLAG-empty vector (EV), FLAG-NUP107-tagged Wilde type (WT), S37A, D831A, Y889C, or ΔN140 vector using Turbofect Transfection Reagent (Thermo Fisher Scientific). Transfected cells were washed with 1XPBS and harvested by scraping after 24 hours. Cells were centrifuged at 3000 rpm for 4 minutes and lysed in a mild NP40 buffer (0.5% NP40, 100 mM NaCl, and 500 mM Tris-HCl pH 7.4) with Protease Inhibitor Single-Use Cocktail (Thermo Fisher Scientific) and Phosphatase Inhibitor (APExBIO) for 20 minutes while being kept on ice. Samples were then centrifuged at 14000 rpm at 4℃ for 20 minutes to pellet cellular debris. Samples were then rotated for 10 minutes at 4℃ and centrifuged at 12000 rpm at 4℃ for 10 minutes. 10% of the volume of the remaining supernatant was taken as an input and stored at -20℃. The remaining samples proceeded to immunoprecipitation and were incubated with anti-Flag M2 affinity beads (Sigma-Aldrich) for 3 hours while rotating at 4℃. After incubation, samples were spun down at 3,000 rpm for 1 minute. The beads of each sample were subsequently washed with ice-cold 0.5% NP40 buffer 3 times. After washing, all samples including the inputs were resuspended with equal amounts of 2x Laemmli buffer followed by boiling at 95℃ for 5 minutes and 1 minute spin at 14,000 rpm before proceeding to SDS-PAGE. A procedure for mass spectrometry is described in Supplementary Methods and Materials.

*Clonogenic survival assay*

HCT116 cells stably expressing pBABE-HA-ER-I-PpoI were seeded into 6-well plates (4000 cells per well) and transfected with the indicated siRNAs for 48 hours, then treated with the 4-OHT with indicated concentrations for 16 hours, then allowed to grow for 10~ 14 days. The plating efficiencies (the number of cells that survive in the absence of drug treatment) were roughly equal between the groups. The cells were fixed with 10% methanol, 10% acetic acid solution for 15 minutes at room temperature. After crystal violet staining, the cells were dissolved with Sorensen buffer (0.5M sodium citrate, 50% ethanol), then the colorimetric intensity of each solution was quantified using Gen5 software on Synergy 2. Error bars are representative of 3 independent experiments.

*Cell cycle analysis*

HeLa and HCT116 cells were transfected with indicated siRNA for 48 hours. Harvested cells were treated with RNase A (200μg/ml) and incubation at 37℃ for 1 hour, then propidium iodide was added to the cell (10μg/ml). Samples were loaded to NovoCyte Flow Cytometer (Agilent technology). Gating and data analysis were performed using the Novoexpress software. For γH2AX intensity analysis, cells were incubated with mouse-anti-γH2AX for 1h and stained with goat-anti-mouse Alexa488 for 30minutes at room temperature.

## Results

#### Polycomb body formation by PHC2 is required to induce transcription repression around DSBs

BMI1 and RNF2 are subunits of the PRC1 complex, which polymerizes and phase separates to form a Polycomb body [21]. Although the presence of BMI1 and RNF2 at DSBs suggests that they have a direct role in the DSB metabolism, whether integrity of the PRC1 complex and its other constituents, are required for the process is unknown. PHC2, a member of PRC1, is required for the formation and clustering of PRC1 multimers and Polycomb body formation [19]. To investigate the involvement of Polycomb body in the DSB metabolism, HeLa cells were irradiated with UVC (254nm) through microfilter, which confirmed the presence of BMI1 at γH2AX foci (Fig 1A). We found that enrichment of BMI1 foci is no longer seen around γH2AX spots upon PHC2 depletion, suggesting that PHC2 is required for the BMI1 localization to DSBs. Since Polycomb component (e.g. BMI1) is implicated in transcription repression at DSB sites[22], we wondered if Polycomb body formation by itself promotes transcription repression at DSB regions. To do this, we depleted PHC2 in the pTurner263 cell line in which we can measure the transcriptional activity near DSBs by targeting the mCherry-Fok1 fusion nuclease upstream of the reporter gene in an inducible manner (Fig 1B) [23]. Consistent with the known role of ATM [8], depleting ATM led to an increase in the YFP-MS2 reporter activity at DSB regions (Supplementary Fig 1). Depleting PHC2 with 4 different siRNAs also led to similar increases in the level of transcription of the YFP-MS2 reporter (Fig 1B). BMI1 or RNF2 was compared as controls, which had similar effects on the reporter activity. This phenomenon was similarly observed when DSBs are induced using UVC microfilter method; while  staining of an elongated form of RNA polymerase II (RNAPII) (p-Ser2) is excluded from the γH2AX regions in control cells, which suggests a suppression of transcription near DSBs, the exclusion is lost when PHC2 was depleted (Fig 1C). BMI1 depletion was compared as a control. We were able to detect the PHC2 protein at the Fok1-induced DSB sites (Fig 1D) and at UVC-induced γH2AX region (Fig 1E). These results suggest that PHC2 also directly participates in the DSB-induced transcriptional repression process, and that the PRC1 complex may contribute to the DSB-induced transcriptional repression.

### Nucleoporins promote transcription repression around DSBs

To better understand how the Polycomb body contributes to the gene silencing around DSBs, we immunoprecipitated FLAG-tagged PHC2 and analyzed the eluate with mass spectrometry (Supplementary Fig 2). Among the notable proteins found in addition to the PRC1 members were Cohesin-associated proteins. PRC1 and Cohesin are known to interact functionally at transcriptionally active loci [24]. We also noted that several members of NPC components are present. Given the previous connection reported between Polycomb and NPC [25], we decided to investigate the role of NPC components further. We noted that many of the nucleoporins co-purified, including NUP107, are part of the Y-complex members of the NPC (Fig 2A). Nup84 in yeast *S. cerevisiae* (orthologue of human NUP107)[26-30] and Nup107 in *drosophila*[31] have been characterized as sites of DSB tethering and repair, yet its function in DSB metabolism in mammalian cells is not well understood. To first confirm the interaction between NUP107 and the Polycomb members, we introduced a 3xFLAG tag into the N-terminus of NUP107 in its genomic loci in HeLa cells using a CRISPR-Cas9 method (Fig 2B). Anti-FLAG immunoprecipitation showed enrichment of BMI1 and PHC2, suggesting that the Polycomb members associate with NUP107. We proceeded to test the possibility that NUP107 and the Y-complex members participate in the process of DSB-induced transcription repression. Interestingly, individual knockdown of the Y-complex nucleoporins including NUP107 (3 independent siRNAs), NUP133, and NUP43, increased the YFP-MS2 reporter expression at Fok1 sites, while knockdown of non-Y complex members such as NUP93 or NUP50 did not (Fig 2C). This trend does not seem to be exclusive to the Y-complex members however, as knockdown of NUP98 or NUP153, non-Y complex members, also had effects on YFP-MS2 expression. NUP153 can impact the integrity of the Y-complex members [32], raising a possibility that

NUP153 may affect the YFP-MS expression indirectly via affecting the Y-complex members. These effects were observed in the UVC assay, as depleting NUP107 led to an overlapping staining of RNAPII (P-ser2) at the γH2AX area (Fig 2D). A similar pattern of transcriptional de-regulation was observed in RPE-1 cells (Supplementary Fig 3) and when DSBs were introduced via I-Ppo1 induction in HCT116 cells (Supplementary Fig 4). The nascent transcription output at the γH2AX can be measured by labeling the cells with 5-EU (5-Ethynyluridine). Knocking down NUP107, NUP43, or NUP133 increased 5-EU intensities along microirradiation-induced γH2AX regions, consistently suggesting an increased transcriptional activity around DSBs (Fig 2E). We previously found that transcriptional de-repression at DSBs is mediated by FACT histone chaperone, which promotes RNAPII elongation via re-organizing nucleosomes [22]. We suggested that the restriction of FACT-dependent transcription is in a linear pathway with BMI1-RNF2 [22]. Consistent with these findings, knocking down the FACT subunit SPT16 completely reversed the transcriptional repression in NUP107-depleted cells (Fig 2F) - this finding suggests that FACT-mediated transcription is aberrantly increased (de-repressed) upon NUP107 depletion. Altogether, these results suggest that nucleoporins, particularly the members of Y-complex, are required for the transcriptional repression around DSBs.

#### Nucleoporins localize to DSB sites

Next, we wished to know whether the nucleoporins localize to DSBs. We found that NUP107 staining is enriched at UVC micropore filter spots and Fok1 spots (validated using two different antibodies against NUP107; (Figs 3A and 3B, respectively). The presence of NUP107 at DSB sites was also confirmed by detecting FLAG-NUP107 protein by anti-FLAG antibody (Fig 3C). NUP43 was also visible at UVC or Fok1-induced DSBs (Figs 3D and 3E, respectively). These results raised an intriguing possibility that these nucleoporin-covered DSBs are present at nuclear periphery. We tracked the NUP107-positive Fok1 sites in time course analysis from the time of 4-OHT/Shield1 treatment, coupled to 3-dimensional (3D) imaging of the cells. The images shown are representative slices from the 3D imaging. In all points analyzed, we could not see the overlapped staining of the NUP107-positive Fok1 with nuclear periphery (stained with Lamin A; Fig 3F). Similar analysis with 53BP1-positive Fok1 spots did not show overlap with nuclear periphery either (Supplementary Fig 5). These results altogether suggest that Y-complex nucleoporins NUP107 and NUP43 localize to DSBs, but the locations of these sites are distant from the nuclear periphery.

#### Interdependency of nucleoporins and Polycomb proteins at DSBs

We next wished to understand whether nucleoporins and Polycomb proteins require each other's presence at DSBs. Firstly, depleting NUP107 or NUP133 reduced the staining of PHC2 at FokI-induced DSB site (Fig 4A). We found that the PHC2 foci formation also depends on ATM. Similar patterns followed for the PHC2 foci formation at γH2AX spots (Fig 4B), where depleting NUP107 or BMI1 reduced the PHC2 foci (Fig 4B). Further, microirradiation-induced recruitment of FLAG-PHC2 (Fig 4C) or FLAG-BMI1 (Supplementary Fig 6) was significantly reduced by depletion of NUP107 or NUP133. Similarly, recruitment kinetics of GFP-RNF2 at micro-irradiated lesions were delayed by knocking down NUP107 or NUP133 (Fig 4D). These results altogether suggested that Y-complex nucleoporins are required for PRC1 members to localize at DSBs. One important readout of the Polycomb function is the induction of the H2AK119-ubiquitin (H2AK119-ub) mark, which is also present at DSB sites in BMI1/RNF2-dependent manner [12]. This modification is associated with and to be an effector of the DSB-induced transcriptional repression [8]. We indeed found that depleting PHC2 led to a marked decrease in H2AK119-ub (Fig 4E) at Fok1 spots. Interestingly, depleting NUP107, NUP133, NUP43 also reduced the levels of H2AK119-ub at the Fok1 spots, consistently suggesting that the nucleoporins promote recruitment of Polycomb members at DSBs. Conversely, depleting Polycomb members BMI1 or PHC2 reduced the NUP107 foci formation at UVC-induced γH2AX sites (Fig 4F) and Fok1 sites (Fig 4G).

Depleting nucleoporins can impact the overall nucleo-cytosolic trafficking of macromolecules. We do not believe the described phenotypes (e.g. reduction of PHC2 foci at γH2AX by NUP107 depletion) are due to a global reduction in the nucleo-cytosolic trafficking, because the PHC2 nuclear staining is minimally affected under the conditions we used (Supplementary Fig 7). Likewise, nuclear staining of Rpb1 (p-Ser2), RAD51, HDAC1 (Supplementary Figs 8, 9, 10 respectively) was not visibly affected by NUP107 knockdown. If any, NUP107 knockdown increased the foci formation of GFP-53BP1 (Supplementary Fig 11) or endogenous 53BP1 (Supplementary Fig 12), in an ATM-dependent manner. These results suggest that NUP107 depletion causes activation of the ATM-53BP1 signaling. Altogether, these results suggest that the recruitment of NUP107 and Polycomb proteins require each other's presence.

### Nucleoporins promote HR repair and genome stability.

DSB-induced gene silencing is generally thought to be a pre-requisite for efficient DSB repair. In support of this notion, various studies found that BMI1 and RNF2 deficiency cause increased genomic instability. We therefore performed a series of experiments to test whether nucleoporins promote DSB repair and genomic stability. First, knocking down NUP43, NUP107, or NUP133 caused marked increases in γH2AX foci formation, similar to BMI1 or PHC2 knockdown (Fig 5A and B). Depleting NUP107 caused a delayed resolution of γH2AX foci after UVC irradiation (Fig 5C), I-Ppo1 induction (Fig 5D), suggesting that DSB repair is hampered. BMI1 and RNF2 were previously shown to regulate RAD51 recruitment and HR repair[33,34]. Indeed, RAD51 foci formation at UVC-induced γH2AX (Fig 5E) and Fok1 (Fig 5F) sites were reduced upon BMI1 or PHC2 depletion. Knockdown of BRCA2, a RAD51 recruiter, was used as a positive control. In both cases, RAD51 foci were markedly reduced upon knockdown of NUP107, NUP133, or NUP43. In further suggesting the role of NUP107 in HR, NUP107 co-localizes with RAD51 at DSB (Fok1) sites (Supplementary Fig 13). At least some of these DSBs positive with NUP107 and RAD51 are in transcriptionally active area, as NUP107 also co-localizes with RAD51AP1 (Supplementary Fig 14), a

RAD51 co-factor that promotes HR repair by promoting DNA-RNA hybrids at transcribed regions[35]. Knockdown of NUP107 reduces the RAD51AP1 foci formation at Fok1 sites (Supplementary Fig 15), consistently suggesting that NUP107 promotes the HR repair. Consistently, knockdown of these nucleoporins reduced the HR frequencies in a cell-based HR assay (Fig 5G; DR-GFP). Further, depleting nucleoporins increased cellular sensitivity to DSB induction via I-Ppo1 induction (Fig 5H). These results altogether suggest that depleting the Y-complex nucleoporins causes HR repair impairment, DSB formations, and increased cellular sensitivities to DSBs.

### ATM/ATR-induced phosphorylation of NUP107 controls DSB-induced silencing

We previously reported that the UBR5-OTUD5 complex antagonizes FACT-mediated transcription around DSBs [17]. Uncoupling the FACT histone chaperone from OTUD5 (by knocking-in a point mutation in the OTUD5 genomic loci; *OTUD5*$^{D537A}$) induces R-loop-associated replication stress and DNA damage [36]. To investigate the factors or pathways becoming activated upon transcription-induced DNA damage stress, we performed a phosphoproteomic analysis of the lysate from *OTUD5*$^{D537A}$ mutant cells, focusing on the SQ/TQ motifs, the target motifs of the ATM/ATR kinases. The analysis identified that the S37 residue of NUP107 is phosphorylated (Fig 6A). This residue was also identified as an ATM/ATR-target site in a phosphoproteomic screen[37], but its functional implication is unknown. We noted that the residue is evolutionarily conserved, especially among higher eukaryotes (Fig 6B). Anti-phospho-SQ/TQ antibody detects the phosphorylation of the immunoprecipitated NUP107 from UVC-irradiate cells, which is undetectable when NUP107 is depleted (Fig 6C), or when the S37 residue is replaced by Ala (S37A) (Fig 6D). Inhibiting either ATM or ATR reduced the p-SQ/TQ signal in immunoprecipitated NUP107 (Fig 6E), suggesting that both ATM and ATR are involved with phosphorylating the residue. We next wished to know if the phosphorylation at the S37 residue influences the DSB-induced transcriptional repression. Interestingly, while transiently expressing the 3xFLAG-NUP107$^{WT}$ partially restored the transcription repression in NUP107-depleted cells, the NUP107$^{S37A}$ mutant could not restore the phenotype (Fig. 6F). Further, H2AK119-ub was partially restored by re-expressing the NUP107 $^{WT}$ but not at all by the NUP107$^{S37A}$ mutant. Quantifying the H2AK119-ub signals enriched in γH2AX spots show that they are much smaller in sizes with weaker intensities in NUP107$^{S37A}$ – expressing cells compared to the wild type counterpart (Fig 6G). These results suggest that the S37A mutation affects the Polycomb-mediated gene silencing around DSBs. Next question we had was whether the S37A mutant fail to support the HR repair capacity. Interestingly, re-expressing the 3xFLAG-NUP107$^{S37A}$ mutant could not restore the HR repair activity in the NUP107-depleted cells, while the 3xFLAG-NUP107$^{WT}$ partially restored (Fig 6H). Expression levels of RAD51 or Polycomb proteins are unchanged in the 3xFLAG-NUP107$^{S37A}$ -expressing cells (Fig 6I). The S37A residue is located within the N-terminal disordered region of NUP107 where no function has been assigned so far, to our knowledge. As disordered regions often serve as platform for protein-protein interactions, we considered that the N-terminal 140 residues that constitute the disordered segment, as predicted by PONDR score (Supplementary Fig. 16), may mediate the association with Polycomb proteins. Indeed, co-IP experiment showed that while 3xFLAG-NUP107$^{WT}$ associates with BMI1 and PHC2, 3xFLAG-NUP107$^{\Delta N140}$ mutant could not to the same extent (Fig 6J). The NUP107$^{\Delta N140}$ mutant could restore neither transcriptional repression (YFP-MS2; Fig 6K) nor foci formation of H2AK119-ub at FokI-induced DSB compared to the wild type counterpart (Fig6L). Thus, the N-terminal 140 residues has functions in DSB repair as NUP107$^{\Delta N140}$ mutant shows retardation in DNA repair (Fig 6M), highlighting the importance of the N140 residues in maintaining genome stability. Altogether, these results suggest that the NUP107 phosphorylation at S37 residue or N140 disorder region contributes to the transcriptional repression at DSBs, possibly via enhancing the interaction with Polycomb protein members.

#### Y-complex integrity is necessary for the transcription repression at DSBs

Since our data suggest a role of the Y-complex members in the DSB-induced transcription repression, we wondered whether maintaining the integrity of the Y-complex is necessary for such function. To address this question, we introduced point mutations within NUP107 (D831A and Y889C) respectively, which are associated with nephrotic syndromes and reduce the interaction between NUP107 and NUP133 (Fig 7A) [38,39]. Of note, Y889 residue is located within the C-terminus region of NUP107 that interacts with NUP133 [40]. We confirmed that these mutations indeed reduce the interaction between NUP107 and NUP133 (Fig 7B) compared to wild type counterpart. Interestingly, NUP107-depleted cells reconstituted with these mutants failed to support the transcription repression at DSBs (Fig 7C), and the H2AK119-ub (Fig 7D). These results argue that the integrity of the Y-complex is necessary for the transcription repression at DSBs.

### Discussion

Herein, we describe a new role of nucleoporins in genome stability maintenance. We found that PHC2, a PRC1 subunit, is required for transcription repression around DSBs. Proteomic analysis linked PRC1 to nucleoporin proteins. Depleting PHC2 or nucleoporins also led to a mis-regulation of transcription repression around DSBs, suggesting that PRC1 and nucleoporins functionally cooperate at DSBs. Consistently, depleting PHC2 or NUP107 decreased the levels of H2AK119-ub, a gene repressive mark induced by PRC1, at DSBs. We found that the localization of nucleoporins and PHC2 at DSBs is dependent on each other. Consistent with the notion that the transcription repression at DSBs facilitates DSB repair, depleting nucleoporins compromised HR repair activity and genomic stability. Importantly, we found that ATM/ATR-mediated phosphorylation of NUP107 at S37 residue is a critical mediator, as loss of this modification leads to transcriptional de-repression and reduced H2AK119-ub at DSBs, concomitant with reduced HR repair activity.

Nucleoporin joins a growing list of factors that regulate transcription around DSBs. Studies suggest that DSB-induced transcription repression is impacted by chromatin remodeling factors, such as Cohesin[9],

PBAF[12], Polycomb epigenetic silencers[12,41]. DITR is also influenced by histone modifiers NuRD[14], KDMA5[42], FACT histone chaperone regulators UBR5-OTUD5[17,22], RNAPII elongation regulators NELFE [15], ENL [11], DYRK1B [16]. ATM may serve as a master regulator across several processes, which phosphorylates PBAF [12] or ENL [11], which ultimately promote Polycomb-mediated transcription repression at DSBs. Our study provides a new link between ATM and Polycomb, by demonstrating that lack of NUP107 phosphorylation at S37 residue reduces the H2AK119-ub mark around DSBs. These results suggest that the NUP107 phosphorylation promotes repressive compartment around DSBs.

Our study provides a new link between transcription repression and an emerging concept of DSB relocation to nuclear periphery. Studies have found that DSBs in certain genomic locations (difficult-to-repair, persistent, or those within heterochromatin) relocate to nuclear periphery (reviewed in [3]). For example, expanded CAG repeats in budding yeast relocate to nuclear periphery in a Nup84-dependent manner (yeast orthologue of NUP107)[43]. DSBs in *Drosophila* pericentromeric heterochromatin are mobilized to nuclear periphery [44] – this relocalization requires nucleoporins including Nup107 and Nup160 - wherein HR repair is ensued [31]. An interesting study using a separation function Nup153 mutation in *Drosophila* showed that the DSB relocation is independent of Nup153, yet still dependent on Nup107[45], suggesting a division of labor among the NPC subunits. DSBs in human cells exhibit increased mobility to form clusters [46], and stalled forks in yeast and human cells are relocalized to periphery [47-50]. On the other hand, a report showed that DSBs in human cells are positionally stable [51]. A recent study showed that rather than relocalizing DSBs to periphery, human cells invaginate nuclear envelope inwards to capture DSBs using kinesins and microtubules as mobilizing forces[7]. A newer study in preprint found that DSBs at transcribed regions are occupied with NE components (doi: https://doi.org/10.1101/2023.05.11.540338).

Although our imaging analysis (Fig 3F) did not suggest that DSBs generated at the LacO array (~4kb upstream of the promoter) overlap with the nuclear periphery, it is possible that our imaging resolution did not capture the membrane components that enables the nucleoporins at DSBs. On the other hand, it is possible that the Y-complex nucleoporins detach from the NE-embedded NPC and exert an independent function at DSBs. More specific separation-function mutants of nucleoporins, such as *NUP107*$^{S37A}$ described herein, should be helpful to further delineate the possibilities. Our findings also suggest that NPC components provide an HR-permissive environment for transcriptionally active chromatin. Similarly, Polycomb members such as BMI1 and RNF2 promote HR repair [10,33,34,41]. Thus, the HR-promoting ability of NPC (Y-complex) and Polycomb members may be co-opted at transcriptionally active area, in cooperation with nucleoporins.

How do the Y-complex nucleoporins engage in transcription repression around DSBs? Emerging evidence point that some nucleoporins function at non-peripheral chromatin independently from their association with NPC. Indeed, the Y-complex was previously shown to exist as a separate pool inside the nucleus[52]. In this vein, we view a possibility that the Y-complex members promote recruitment of Polycomb silencers to non-peripheral DSBs. Cooperation of these factors may promote formation of a chromatin domain that is inaccessible by RNAPII (Fig 8 model). Previous studies have reported a link between nucleoporins and Polycomb, where NUP153 recruits PRC1 components to developmentally regulated genes to keep them in repressed states in mouse embryonic stem cells [53]. In the study, they noted that the role of NUP153 is not linked to the nuclear import of PRC1, rather NUP153 interacts with PRC1 components and co-occupy transcription start sites of selected genes, suggesting a direct role in chromatin regulation separate from NPC. Another study using HeLa cells found that NUP153 interacts with Cohesin subunits and co-occupy TSS of immediate early genes, where NUP153 keep them in repressed states[54]. They found that one of the genes upregulated upon NUP153 depletion was *Hoxb13*, a Polycomb-responsive gene, providing another clue that nucleoporins and Polycomb silencers may cooperate. A study on chromatin mapping of nucleoporins in *Drosophila* cells found that Nup93 associates with Polycomb-silenced regions while Nup107 preferentially targets active genes [25]. We did not find NUP93 to be critical to DSB-induced silencing (Fig 2C), although NUP93 may functionally interact with Polycomb in other aspects of gene regulation in human cells. Multiple studies showed that the Y-complex members of *S. cerevisiae*, such as Nup84 or Nup133, is required for radioresistance and promotes recombination repair[27,29,55-57]. We consistently found that depleting NUP107 impairs HR, and highlighted that at least two components are required in the process; ATM/ATR-induced phosphorylation at S37 residue of NUP107, and ability of NUP107 to form complex with NUP133. Although our study highlights the Y-complex members, whether other nucleoporins also contribute to this process remains a possibility. For instance, NUP54 [58] and NUP153 [59-61] contributes to genomic stability but its involvement in DSB-induced transcription repression is unknown. Depleting NUP153 also caused transcription de-repression (Fig 2C), but this effect could be due to disruption of the NPC integrity [32]. As PBAF complex cooperates with the PRC1 complex in DSB-induced silencing[12], it is also possible that nucleoporins may influence functionality of the PBAF complex. Further studies may enlighten whether such cooperative regulation exists. In sum, our study begins to decipher the role of Polycomb body and nucleoporins in DSB metabolism, DSB repair, and genome maintenance.

## Declarations

#### Data Availability

The datasets generated during and/or analyzed during the current study are available from the corresponding authors on reasonable request.

#### Acknowledgement

We thank Bin Fang at the Proteomics and Metabolomics Core at the H. Lee Moffitt Cancer Center & Research Institute for proteomic analysis and WASHU Genome Engineering & iPSC center for generating all CRISPR cell lines. We thank Roger Greenberg for the pTuner 263 cell line, Maria Jasin for the DR-GFP

cell line, and Michael Kastan for pBABE-HA-ER-I-Ppo1 plasmid through Addgene. We also thank Hongtae Kim for helpful discussions.

#### Funding

This work was supported by National Institutes of Health (R01GM117062-01A1), National Research Foundation of Korea (NRF-2021R1A2C1093818), and DGIST R&D Program of the Ministry of Science and ICT (23-HRHR-05) to Y.K. The work was also supported by IBS-R022-D1 to K.M and NIH R35-GM136250 to M.P. (MP is an investigator with the Howard Hughes Medical Institute).

#### Author Contributions

H.S., Y.B., S.K., D.D. performed most of the experiments. G.R. and E.L. performed the laser-micro-irradiation experiments. Y.L., S.L., S.K. supported the immunoprecipitation and imaging analysis. Y.K., K.M, and M.P. supervised the experiments. All authors have contributed to the designing and interpreting of the experiments. Y.K. conceived the overall studies and wrote the manuscript, and all other authors have critically reviewed.

#### Competing interests

M.P. is or has been an advisor for and has financial interests in SEED Therapeutics, Triana Biomedicines, and CullGen, Kymera Therapeutics, and Umbra Therapeutics. The other authors have no competing interests to declare.

## Figures

Figure 1

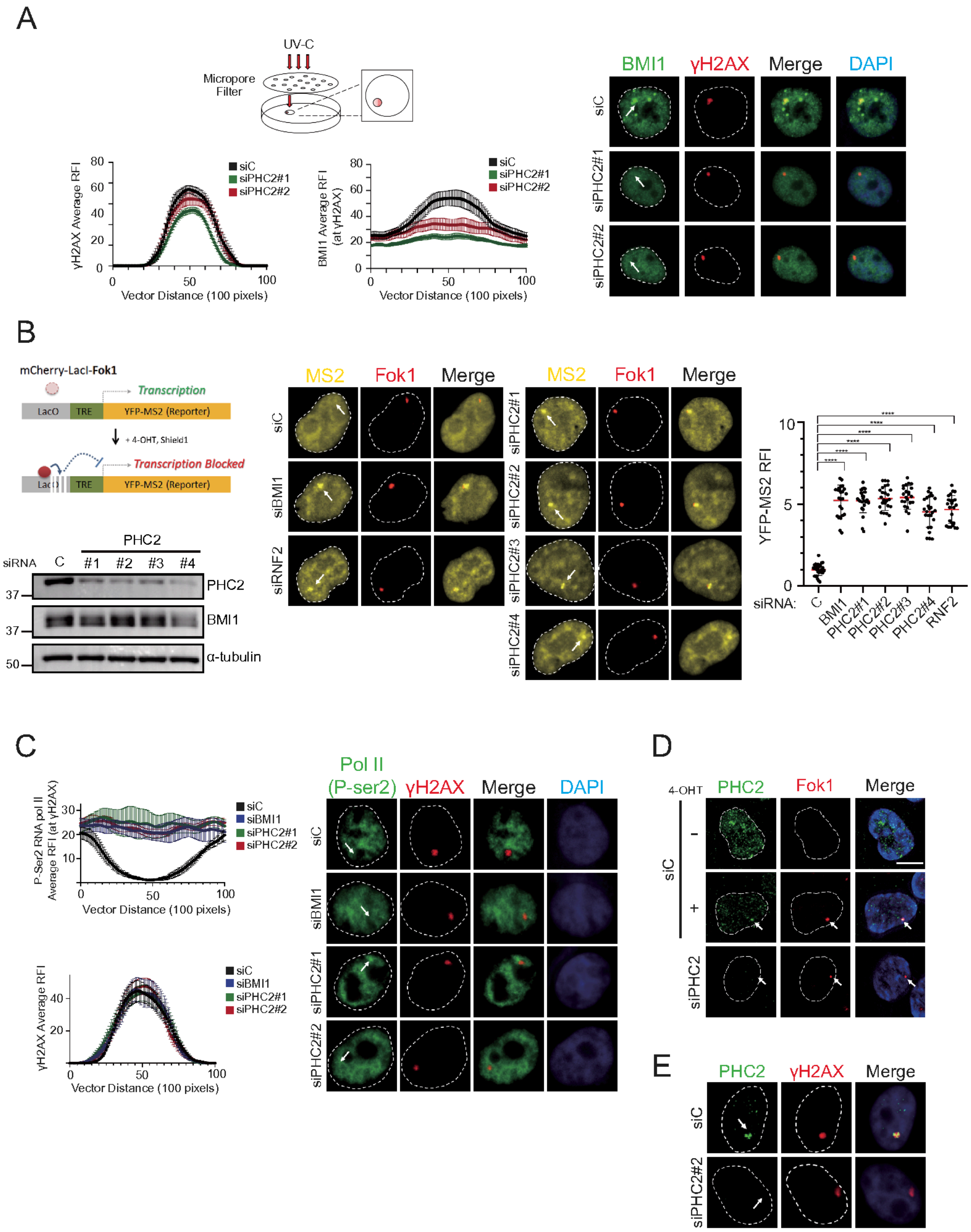

Figure 1

### PHC2 regulates transcription around DSBs.

(A) (Top) Schematic of cell irradiation by UV-C through a Micropore filter. (Right) BMI1 foci formation was induced in Hela cells by UV-C through a micropore filter (100 J/m2; 1 hour recovery) following the siRNA treatment. (Left) Quantification of the average RFI of BMI1 and γH2AX along a vector of 100 pixels (n=20).

(B) Schematic of U-2OS based reporter cell line (pTuner263) in which induction of the mCherry-tagged Fok1 endonuclease induces double-strand break in upstream of a doxycycline-inducible reporter gene. Transcription activity is tracked by YFP-MS2 that binds secondary structure in nascent mRNA from the reporter gene. pTuner263 cells transfected with indicated siRNAs, then Fok1 nuclease was induced by 4-OHT and Shield-1, and transcription of the YFP-MS2 reporter was induced by doxycycline 2h before fixing (n=30) (**** indicates P-value < 0.0005). (Left) Western blot confirmation of siRNA.

(C) HeLa cells transfected with indicated siRNAs were locally irradiated with UV (100 $J/m^2$) through micropore filter, fixed in 1h. (Right) representative image. (Left) measurements of the average RFI of RNA polymerase II (P-ser2) along line across the γH2AX spot (n=15).

(D) pTuner263 cell was treated with 4-OHT and Shield-1 for 2h before fixing, then stained with PHC2.

(E) γH2AX overlap with PHC2 in HeLa irradiated with UV through the micropore filter.

Figure 2

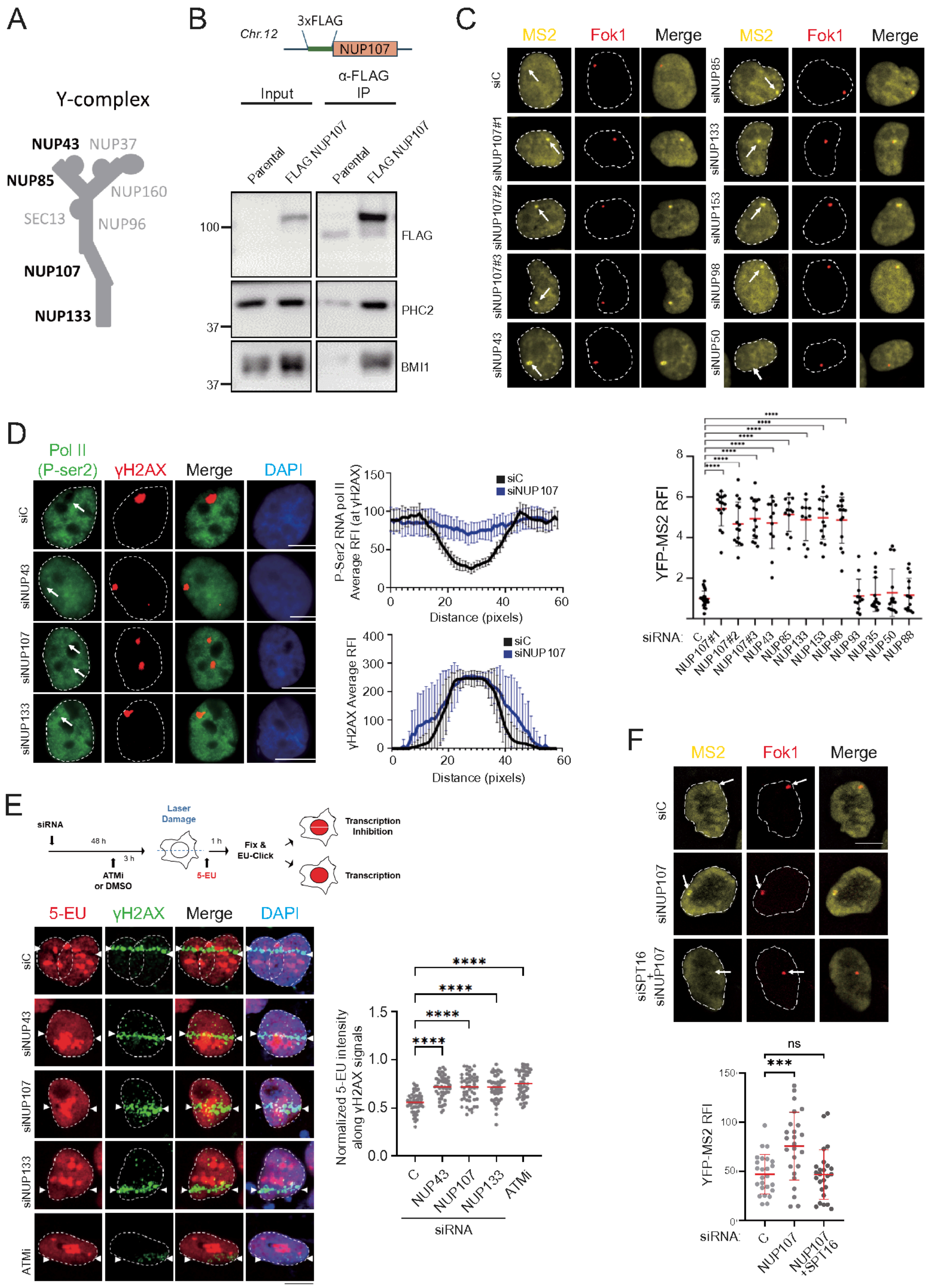

## Figure 2

### Nucleoporins regulate transcription repression around DSBs.

(A) Schematic of structure and components of Y-complex nucleoporin.

(B) anti-FLAG immunoprecipitation blotted with indicated antibodies in FLAG-NUP107 CRISPR-Cas9 KI HeLa cell.

(C) Transcription repression was monitored in pTuner263 cell treated with indicated siRNAs targeting nucleoporins (n=15, **** indicates P-value <0.0005).

(D) The γH2AX overlap with Pol II (P-ser2) in HeLa transfected with indicated siRNAs for 48 h, then irradiated with UV (100 J/$m^2$) through the micropore filter, 1 hour before fixing. (Left) Representative images. (Right) Measurement of the average RFI along lines across the γH2AX spot (n=10).

(E) (Top) Experimental schematic of transcription tracking after micro-irradiation. Briefly, U-2OS cells were transfected with corresponding siRNAs for 48 h and treated with either ATM inhibitor (KU-60019, 10 mM) or DMSO for 3hours before irradiation. After micro-irradiation, cells were incubated with 100 mM EU for 1 h, then fixed and subjected to a click reaction. (Bottom) Representative images of 5-EU (5-Ethynyl Uridine) and gH2AX staining. The normalized mean intensity of 5-EU at micro-irradiated sites are quantified and plotted (**** indicates P-value < 0.0001).

(F) Transcription repression measured in pTuner263 cell treated with siRNA targeting NUP107 and SPT16. 4-OHT, Shield-1 and Doxycycline were treated for 2h before fixation (n=25 each) (*** indicates P-value < 0.001).

Figure 3

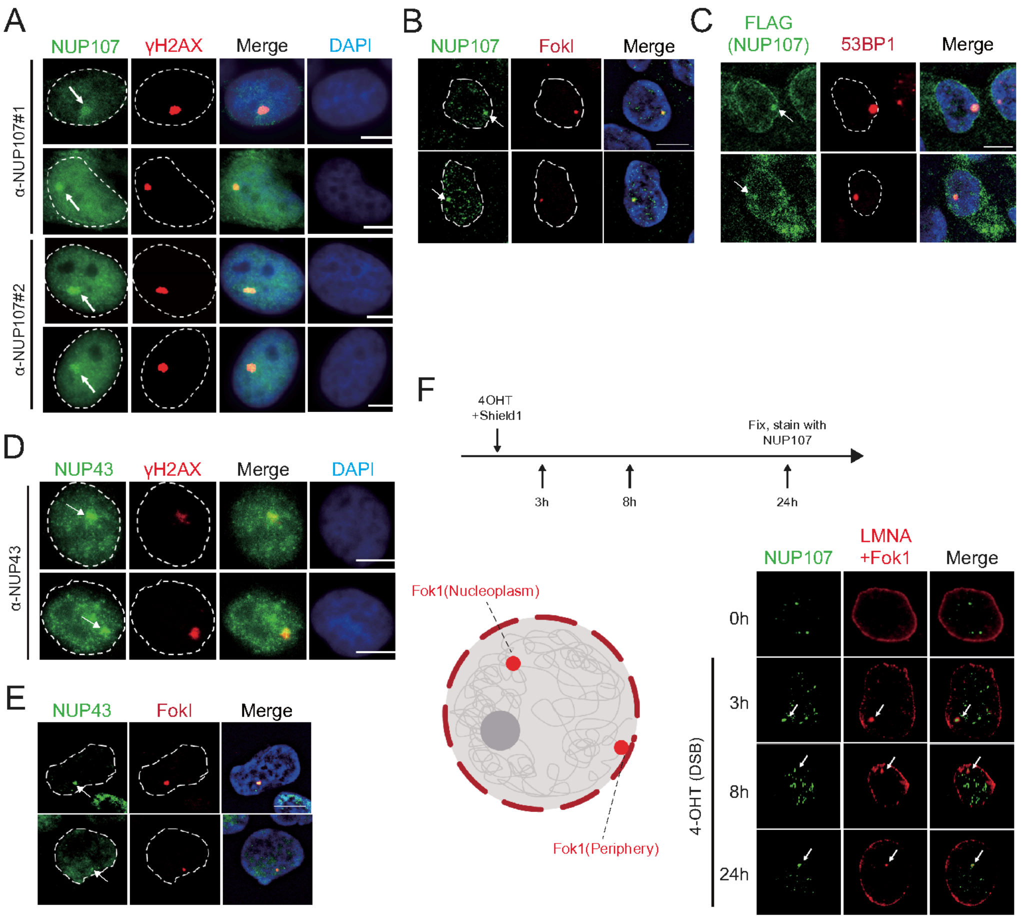

### Figure 3

### Nucleoporins localize to DSBs.

(A, D) HeLa cells were locally irradiated with UV (1 J/m$^2$) through 5 μM micropore filters 1 hour before fixing, then co-stained with γH2AX and NUP107 (two different antibodies) or NUP43.

(B, E) pTuner263 cells were stained with NUP107 or NUP43. 4-OHT and Shield-1 were treated for 2 hours before fixing.

(C) HeLa cells stably expressing FLAG-NUP107 were locally irradiated with UV (1 J/m$^2$) through 0.5 μM micropore filters before fixing, then co-stained with 53BP1 and FLAG.

(F) pTuner263 cells were treated with 4-OHT and Shiled-1 for indicated time. The distance between FokI and Nuclear periphery were measured.

Figure 4-1

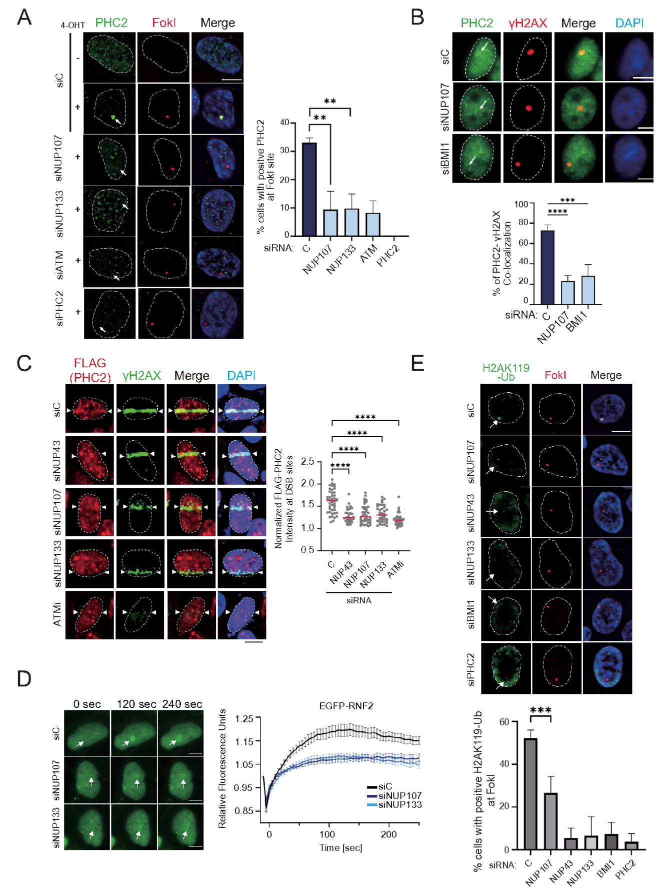

Figure 4-2

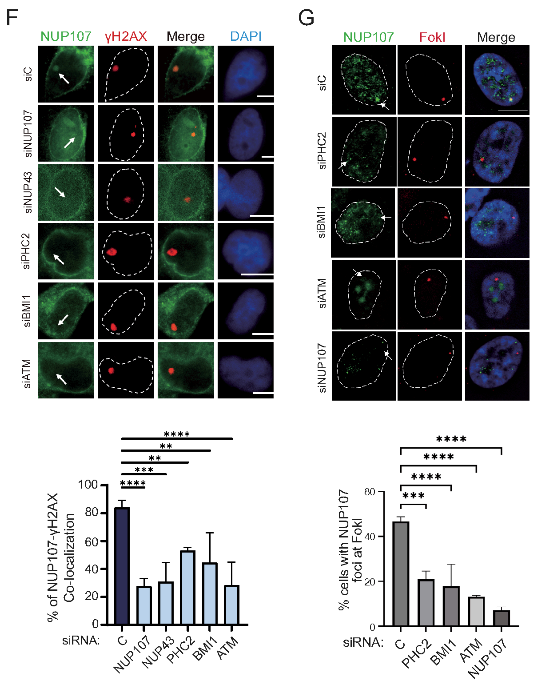

### Figure 4

**Recruitment of nucleoporins and Polycomb proteins to DSBs are interdependent.**

(A) pTuner263 cells were stained with PHC2. Representative images (left) and the measure of overlap between PHC2 and mCherry-FokI (right), the experiments were conducted in triplicate.

(B) HeLa cells transfected with indicated siRNAs were locally irradiated with UV (1 J/m$^2$) through 5 μM micropore filter, fixed in 1h. Then co-stained with PHC2 and γH2AX. (Top) representative image. (Bottom) measurements of the % of PHC2 and γH2AX overlap.

(C) U-2OS cells were transfected with corresponding siRNAs, and cDNAs to express FLAG-PHC2, and subjected to a micro-irradiation. ATM inhibitor (KU-60019, 10 mM) or DMSO before irradiation, and cells were then fixed with and immunostained with FLAG and gH2AX antibodies. (Left) Representative images of FLAG (PHC2) and gH2AX staining. (Right) The normalized mean intensity of FLAG (PHC2) at micro-irradiated sites are quantified and plotted (**** indicates P-value < 0.0001).

(D) U-2OS cells stably expressing EGFP-RNF2 were transfected with siRNAs. Cells were pre-sensitized with BrdU (10 μM) for 24 hours and subjected to 405 nm laser induced damage. DNA damage recruitment dynamics were captured by live cell imaging. Relative fluorescence values and images were acquired every 5 s for 5 min. For each condition, ≥25 cells were evaluated. Mean relative fluorescence values and standard errors were plotted against time.

(E) pTuner263 cells transfected with indicated siRNAs for 48h, then stained with H2AK119-Ub. Representative images (top) and the measure of overlap between H2AK119-Ub and mCherry-FokI (bottom). The experiments were conducted in triplicate (n > 40).

(F) HeLa cells transfected with indicated siRNAs for 72h were locally irradiated with UV ($1J/m^2$) through 5μM micropore filter, fixed in 1h. Then co-stained with NUP107 and γH2AX. (Top) representative image. (Bottom) measurements of the % of NUP107 and γH2AX overlap.

(G) pTuner263 cells transfected with indicated siRNAs for 48h, then stained with NUP107. Representative images (top) and the measure of overlap between NUP107 and mCherry-FokI (bottom). The experiments were conducted in triplicate. (**** indicates P-value < 0.0001, *** indicates P-value < 0.001)

Figure 5

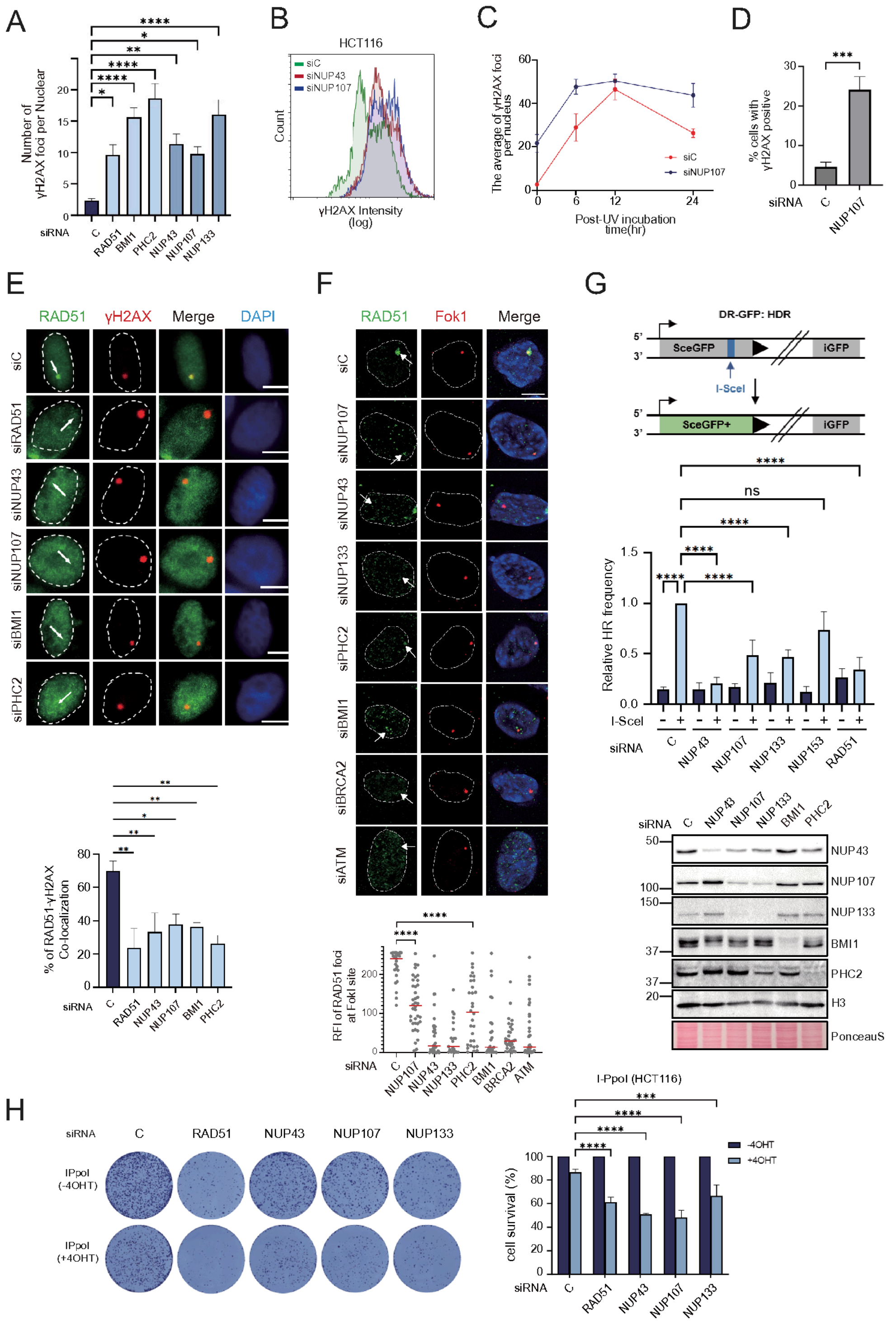

Figure 5

### Nucleoporins promote genomic stability.

(A) The measurement of the average of γH2AX foci. HeLa cells transfected with indicated siRNAs for 72h and irradiated with UVC (1 J/m$^{2}$), then recovered for the indicated time points before fixing (**** indicates P-value <0.0001).

(B) HCT116 cells transfected with indicated siRNAs for 48h were stained with γH2AX. Intensity of γH2AX in log was analyzed by flow cytometry.

(C) The measurement of the number of γH2AX foci. HeLa cells transfected with indicated siRNAs for 72h (n > 100).

(D) The measurement of the average of γH2AX foci. Stably expressing I-PpoI HCT116 cells were transfected with indicated siRNAs for 72h. 4-OHT (1 μM) were treated to the cell for 9 hours, then medium was changed fresh DMEM. Cells were fixed 3 hours after washout. The experiments were conducted in triplicate (n=100).

(E) HeLa cells transfected with indicated siRNAs for 72h were locally irradiated with UV (1 $J/m^2$) through 5μM micropore filter, fixed in 1h. Then co-stained with RAD51 and γH2AX. (Top) representative image. (Bottom) measurements of the % of RAD51 and γH2AX overlap (** indicates P-value < 0.002).

(F) pTuner263 cells transfected with indicated siRNAs for 48h, then stained with RAD51. Representative images (top) and the measure of overlap between RAD51 and mCherry-FokI (bottom). The experiments were conducted in triplicate (n > 25).

(G) (Top) Schematic of homology-directed repair substrate. HR leads to the restoration of GFP by gene conversion (iGFP). (Bottom) U-2OS-DR-GFP cells were transfected with corresponding siRNAs, and homology-directed repair frequency was measured 48 h after transfection with the I-SceI endonuclease. Data in graph represent mean ± SD of at least three independent experiments. Western blot confirmation of siRNA. Statistical analysis: One-way ANOVA, along with a post-hoc Tukey test; **** < 0.0001, ns: non-significant.

(H) Stably expressing I-PpoI HCT116 cells transfected with indicated siRNAs. After 48 hours medium was changed with or without 4OHT (1 μM). (Left) representative image stained with crystal violet. (Right) measurements are as mean ± SD from three different experiments (**** indicates P-value < 0.0001).

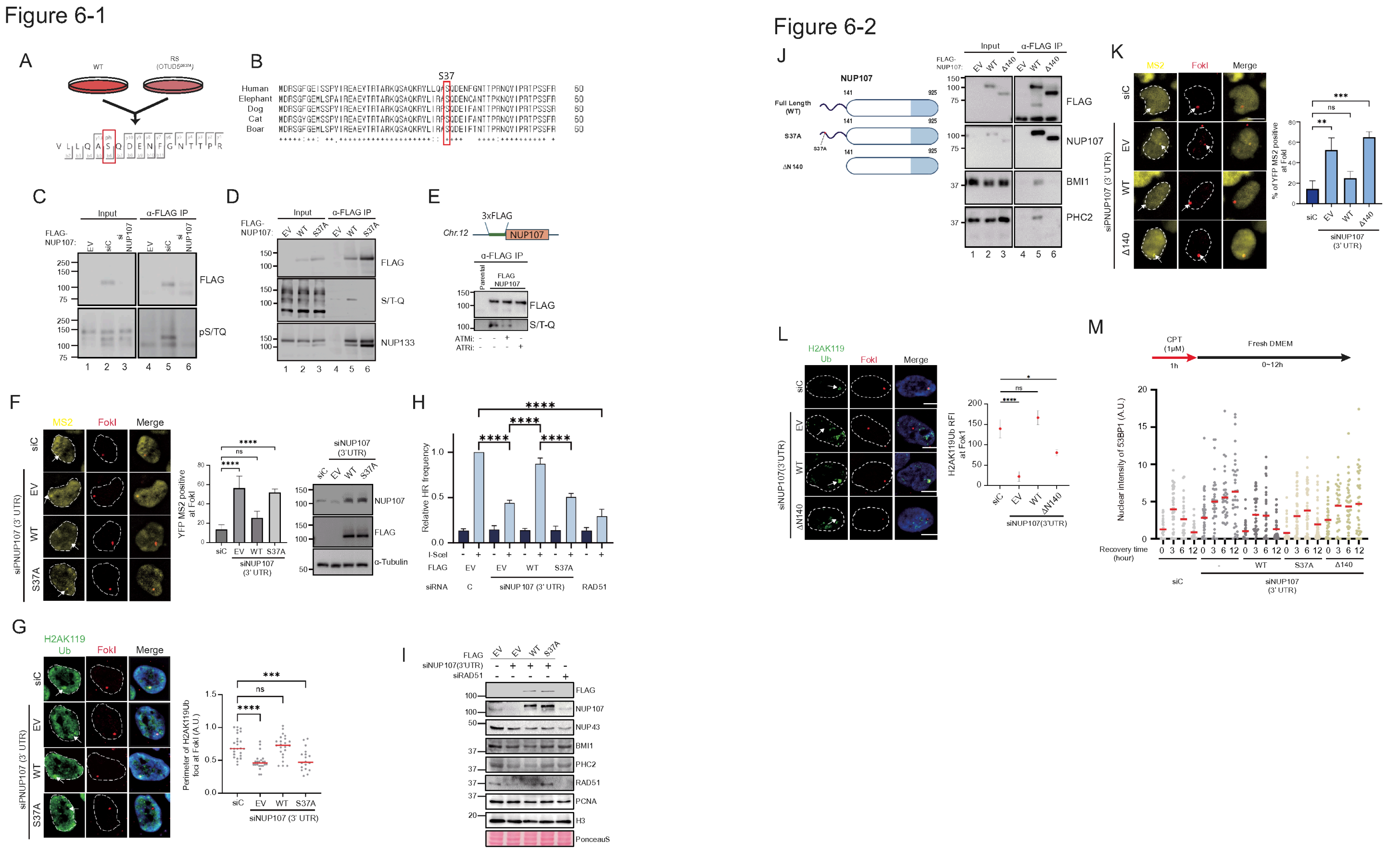

### Figure 6

#### ATM/ATR-mediated phosphorylation of NUP107 contributes DSB-induced silencing

(A) A scheme depicting the identification of phospho-sites using mass spectrometry. Parental HCT116 and OTUD5 CRISPR KI cell were used.

(B) Sequence alignment of phosphorylation site (S37) of NUP107 among species.

(C) HeLa FLAG-NUP107 Knock-In cells were transfected with siRNAs for 72h and treated with 1J/m$^2$ UV followed by incubation for 1h at 37∘C. After cells were lysed, anti-FLAG IP was performed.

(D) 293T cells stably expressing FLAG-NUP107 WT or S37A mutant were treated with 1J/m$^2$ UV followed by incubation for 1h at 37∘C. After cells were lysed, anti-FLAG IP was performed.

(E) FLAG-NUP107 KI HeLa cell and parental HeLa cell were treated with DMSO or ATM inhibitor (KU55933, 20uM) or ATR inhibitor (AZ20, 5uM) for 3hours respectively, then irradiated with UVC (1 J/m$^2$). After 1 hour, cells were harvested and subject to lysis.

(F) pTuner263 cells were transfected with siRNA targeting the 3'UTR region of NUP107, then transfected with indicated NUP107 constructs (EV = empty vector). After 24 hours, the cells were treated with 4-OHT,

Shield-1 and Doxycycline for 2hours. The assay was performed in triplicates (n=100, each). (Statistical analysis: One-way ANOVA, **** indicates P-value < 0.0001).

(G) pTuner263 cells were transfected with siRNA targeting the 3’UTR region of NUP107, then transfected with indicated NUP107 constructs (EV = empty vector). After 24 hours, the cells were treated with 4-OHT and Shield-1 for 2hours. Cells were fixed and stained with H2AK119Ub (n=25, each). (Statistical analysis: One-way ANOVA, **** indicates P-value < 0.0001, *** indicates P-value < 0.001).

(H) U-2OS-DR-GFP cells were transfected with either 3’UTR targeting NUP107 or RAD51 siRNA as indicated. After 6 h siRNA transfection, FLAG-tagged cDNAs expressing empty vector (EV), NUP107 wild-type (WT), and S37A mutant were transfected for 48 h. Homology-directed repair frequency was measured 48 h after transfection with the I-SceI endonuclease. Data in graph represent mean ± SD of at least three independent experiments. (Statistical analysis: One-way ANOVA, along with a post-hoc Tukey test; **** < 0.0001).

(I) Western blot for (H)

(J) (Left) Schematic of NUP107. S37 in disordered region in the N-terminal is indicated. (Right) Western blot of Co-IP. HeLa cells were transiently transfected with indicated plasmids, incubated for 24 hours. Cells were lysed and subjected to FLAG-IP.

(K) Transcription repression at DSB was measured using pTuner263 cell transfected with siRNAs targeting 3’UTR of NUP107 and indicated NUP107 constructs. The experiments were conducted in triplicate (n > 50) (Statistical analysis: One-way ANOVA, **** indicates P-value < 0.0001).

(L) pTuner263 cell transfected with siRNA targeting NUP107 (3’UTR) and indicated FLAG-NUP107 variants were stained with H2AK119-Ub. Representative images indicate the measure of overlap between H2AK119-Ub and mCherry-FokI (n > 15).

(M) HeLa cells stably expressing $NUP107^{WT}$, $NUP^{S37A}$, or $NUP^{\Delta 140}$ were transfected with siRNA targeting endogenous NUP107 (3’UTR). Cells were treated with Camptothecin(CPT) 1uM for 1hour, then recovered, fixed, and imaged with 53BP1 antibodies (n > 20 each).

Figure 7

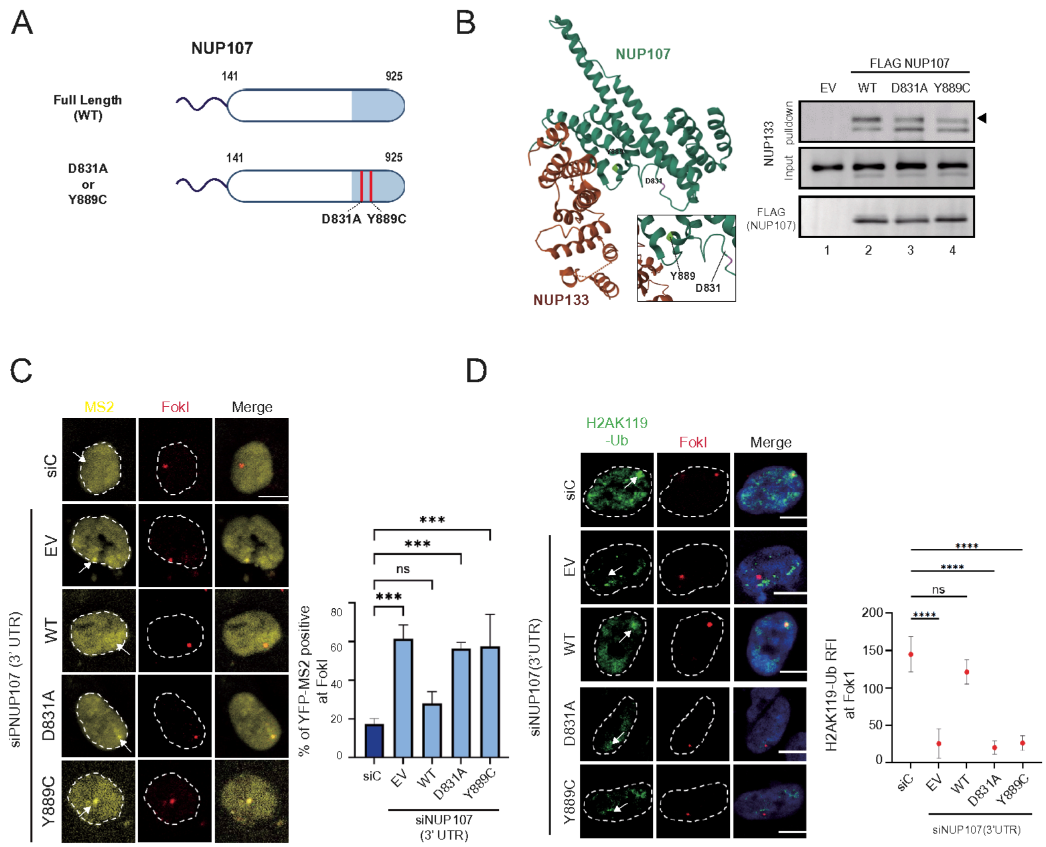

### Figure 7

### Y-complex integrity is necessary for the transcription repression at DSBs

(A) Schematic of NUP107 and its mutant used in the study.

(B) (Left) model of NUP107-NUP133 complex from Protein Data Bank (PDB:3CQC). (Right), Co-immunoprecipitation analysis of FLAG NUP107 WT and its alterations. Indicated NUP107 constructs were transiently transfected in HeLa cells and subject to cell lysis.

(C) Transcription repression measured in pTuner263 cell transfected with siRNAs targeting 3'UTR of NUP107 and indicated NUP107 constructs. The experiments were conducted in triplicate (n > 50 each).

(D) pTuner263 cell transfected with siRNA targeting NUP107 and indicated FLAG-NUP107 variants were stained with H2AK119-Ub. Representative images indicate the measure of overlap between H2AK119-Ub and mCherry-FokI (n > 15 each).

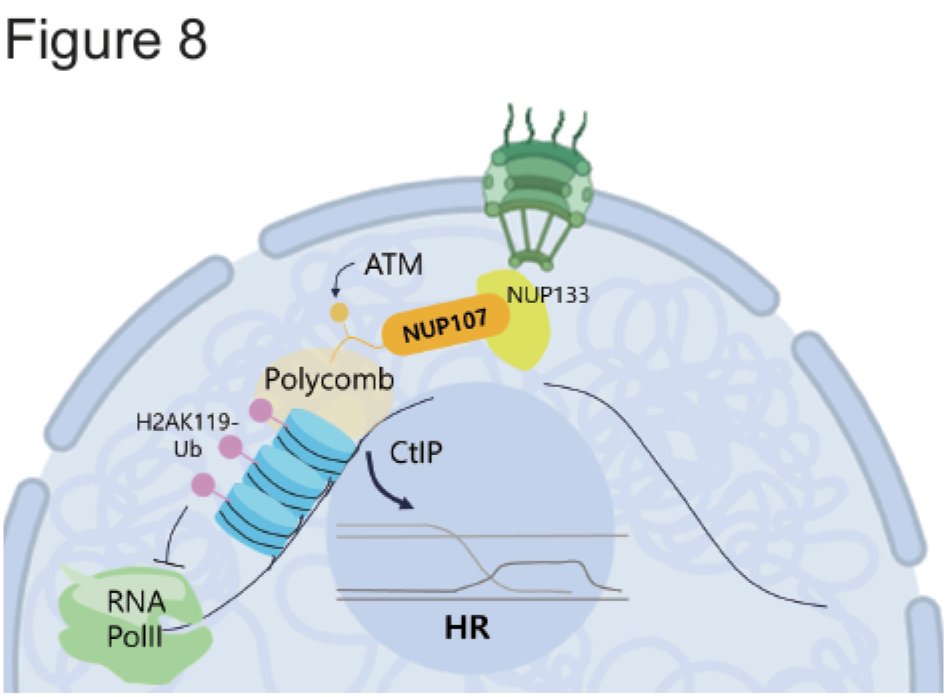

**Figure 8**

**Model for the proposed mechanism.**

(Image was generated using BioRender.)

### Supplementary Files

This is a list of supplementary files associated with this preprint. Click to download.

- SuppleFigcombined.pdf
- Supplementaryinfromation.docx
